# Outcomes from international field trials with Male *Aedes* Sound Traps: Frequency-dependent effectiveness in capturing target species in relation to bycatch abundance

**DOI:** 10.1371/journal.pntd.0009061

**Published:** 2021-02-25

**Authors:** Kyran M. Staunton, Donovan Leiva, Alvaro Cruz, Joelyn Goi, Carlos Arisqueta, Jianyi Liu, Mark Desnoyer, Paul Howell, Francia Espinosa, Azael Che Mendoza, Stephan Karl, Jacob E. Crawford, Wei Xiang, Pablo Manrique-Saide, Nicole L. Achee, John P. Grieco, Scott A. Ritchie, Thomas R. Burkot, Nigel Snoad

**Affiliations:** 1 College of Public Health, Medical and Veterinary Sciences, James Cook University, Smithfield, Australia; 2 Australian Institute of Tropical Health and Medicine, James Cook University, Smithfield, Australia; 3 Belize Vector and Ecology Center (BVEC), Orange Walk Town, Belize, Central America; 4 Vector-Borne Diseases Unit, PNG Institute of Medical Research, Madang, Papua New Guinea; 5 Collaborative Unit for Entomological Bioassays (UCBE) and the Laboratory of Biological Control for *Ae*. *aegypti*, Universidad Autónoma de Yucatán, Merida, México; 6 Verily Life Sciences, San Francisco, California, United States of America; 7 School of Engineering and Mathematical Sciences, La Trobe University, Melbourne, Australia; 8 Department of Biological Sciences, Eck Institute for Global Health, University of Notre Dame, Notre Dame, Indiana, United States of America; Centers for Disease Control and Prevention, UNITED STATES

## Abstract

*Aedes aegypti* and *Aedes albopictus* vector dengue, chikungunya and Zika viruses. With both species expanding their global distributions at alarming rates, developing effective surveillance equipment is a continuing priority for public health researchers. Sound traps have been shown, in limited testing, to be highly species-specific when emitting a frequency corresponding to a female mosquito wingbeat. Determining male mosquito capture rates in sound traps based on lure frequencies in endemic settings is the next step for informed deployment of these surveillance tools. We field-evaluated Male *Aedes* Sound Traps (MASTs) set to either 450 Hz, 500 Hz, 550 Hz or 600 Hz for sampling *Aedes aegypti* and/or *Aedes albopictus* and compared catch rates to BG-Sentinel traps within Pacific (Madang, Papua New Guinea) and Latin American (Molas, Mexico and Orange Walk Town, Belize) locations. MASTs set to 450–550 Hz consistently caught male *Ae*. *aegypti* at rates comparable to BG-Sentinel traps in all locations. A peak in male *Ae*. *albopictus* captures in MASTs set at 550 Hz was observed, with the lowest mean abundance recorded in MASTs set to 450 Hz. While significantly higher abundances of male *Culex* were sampled in MASTs emitting lower relative frequencies in Molas, overall male *Culex* were captured in significantly lower abundances in the MASTs, relative to BG-Sentinel traps within all locations. Finally, significant differences in rates at which male *Aedes* and *Culex* were positively detected in trap-types per weekly collections were broadly consistent with trends in abundance data per trap-type. MASTs at 550 Hz effectively captured both male *Ae*. *aegypti* and *Ae*. *albopictus* while greatly reducing bycatch, especially male *Culex*, in locations where dengue transmission has occurred. This high species-specificity of the MAST not only reduces staff-time required to sort samples, but can also be exploited to develop an accurate smart-trap system—both outcomes potentially reducing public health program expenses.

## Author summary

The continued global expansion of *Aedes aegypti* and *Aedes albopictus*, which transmit viruses causing dengue and Zika, necessitates the further development of effective mosquito traps for monitoring and predicting transmission risk. Mosquito sound traps attract male mosquitoes by producing a frequency which mimics the wingbeat sound of female mosquitoes. As few insects will respond to such sounds, these traps can be highly species-specific, saving significant person-time to sort samples, potentially paving the way for more accurate smart-trap systems and reduced surveillance costs. To date, limited research has occurred within field-settings investigating the capture rates of male mosquitoes in sound traps set to different frequencies. To this point, we performed trials in dengue endemic environments to quantify capture rates of mosquitoes in Male *Aedes* Sound Traps set to different frequencies in Papua New Guinea, Mexico and Belize. We found that both male *Ae*. *aegypti* and *Ae*. *albopictus* responded positively to frequencies between 450 and 600 Hz. Additionally, male *Culex* mosquitoes were also caught, but at lower abundances in traps set to higher sound frequencies. These traps, when set to 550 Hz, effectively capture male *Ae*. *aegypti* or *Ae*. *albopictus* with limited bycatch.

## Introduction

*Aedes aegypti* and *Aedes albopictus* are the two most important vectors of the viruses responsible for dengue, Zika and chikungunya [1–3]. Both mosquito species have expanded their distributions in recent years [4], with *Ae*. *aegypti* projected to continue dispersing into tropical and subtropical regions and *Ae*. *albopictus* spreading more globally, such as within Central [5] and South America [6], the Pacific region [7] and temperate regions in Europe and the United States of America [8]. Efforts to successfully control these mosquitoes requires cost-effective surveillance tools which not only detect mosquitoes in sufficient abundances to monitor population trends, but can regularly detect the presence of mosquitoes throughout the mosquito season with minimal burden to public health programs.

Male mosquitoes are mass-reared for a variety of mosquito control programs utilising *Wolbachia*-infected mosquitoes [9–11] and recently, there has been renewed development of mosquito sound traps mimicking female mosquito wingbeat frequencies [12–16] to attract and capture male mosquitoes. A speaker integrated into a trapping unit encourages male mosquitoes to fly into a capture chamber, removing the need for fans which damage samples during capture [17,18]. Traps incorporating fans are highly effective in securing mosquitoes [19–21], but incur substantial operational costs in long-term adult *Aedes* surveillance programs (e.g., costs of 12 V batteries or mains power, person-time required to sort and identify captured insects). The indiscriminate collection of non-target species by traditional traps impedes the accurate identification of specimens, increases labour to identify samples and poses a significant challenge for the development of mosquito smart-trap systems, which aim to detect and communicate catches to surveillance staff.

Although wingbeat frequencies typically vary between 150–200 Hz (5^th^ to 95^th^ percentile of range) for a single species in natural settings [22], sound traps can fine-tune the sound-lure frequency to one which is differentially attractive for a desired target species group compared to bycatch. *Culex* females produce mean frequencies which generally peak under 400 Hz while female *Ae*. *aegypti* wingbeat frequencies are slightly higher (e.g., can peak around 458–460 Hz) and *Ae*. *albopictus* female frequencies higher still (e.g., can peak between 536–544 Hz) [22–24]. Hence, sound traps deployed to catch *Culex* males have been set to frequencies of 370 Hz or 400 Hz [25–30], while traps targeting *Ae*. *aegypti* used sound lures set to 484–500 Hz [14,18,31] and those investigating the attraction of male *Ae*. *albopictus* were set to 545–649 Hz [12].

Female wingbeat frequencies, and male attraction to these frequencies, are affected by biological and environmental factors, such as male and female body size, age and ambient temperature [27,32–36]. Additionally, male attraction to various wingbeat frequencies can also change due to their mating experience and larval rearing conditions [25,37]. Subsequently, the actual ranges of wingbeat frequencies to which males are exposed, and how males respond to these frequencies, may vary considerably, especially in natural settings where a wider range of wingbeat frequencies may facilitate successful mating [27]. For example, to effectively differentiate mosquito species recorded in the field, Mukundarajan, Hol et al. [22] classified different wingbeat frequencies by distributions varying by up to 100 Hz for each species, rather than specific mean values.

Understanding the extent to which male mosquitoes are attracted to specific frequencies in a natural environment is a vital step towards effective deployment of sound traps for surveillance. While Kahn and Offenhauser [38] first deployed sound traps in the 1940s and Ikeshoji and colleagues performed multiple experiments attempting to control field mosquito populations with sound traps in the 1980s [26,29,30,39], to date, few field studies have assessed male attraction to sound traps set to different frequencies. In the 1980s, Kanda, Cheong et al. [40] surveyed male Malaysian *Mansonia* and Ikeshoji and Ogawa [39] sampled male *Ae*. *albopictus* and *Culex tritaeniorhynchus* in Japan. Recently, Swan, Russel et al. [41] assessed the attraction of male *Ae*. *albopictus* to Male *Aedes* Sound Traps (MASTs) set to fixed frequencies ranging between 450 and 700 Hz in northern Australia. While the effectiveness of these MASTs was not compared to standard mosquito traps, the researchers did find that MASTs set to frequencies between 500 and 650 Hz caught the highest abundances of males in this location. Little field research has otherwise occurred investigating male mosquito attraction to various frequencies, therefore we clearly still have much to learn.

We assessed MAST capture rates of male *Ae*. *aegypti* and *Ae*. *albopictus*, as well as, medically important *Culex* species (*Culex quinquefasciatus*, *Culex restuans* and *Culex nigripalpus*), using sound-lures set to either 450 Hz, 500 Hz, 550 Hz or 600 Hz under natural environmental conditions in three dengue endemic countries (Papua New Guinea, Mexico and Belize). BG-Sentinel (BGS) traps (Biogents, Regensburg, Germany) were integrated into the evaluation design as the gold standard surveillance trap comparator for *Ae*. *aegypti* and *Ae*. *albopictus*) to establish benchmarks. We assessed differences in mean male abundances per trap type, and confirmed these trends against the proportion of positive weekly detections of male mosquitoes to ensure that the trap-types which caught the most abundant male mosquitoes also most frequently detected their presence.

## Methods

### Trap description

The MAST is a low-powered and highly specific water-resistant sound-baited mosquito trap consisting of two main components [18]. The large black base acts as a swarm marker to visually attract male *Aedes* while the clear plastic 2.5 L head houses a speaker attracting mosquitoes that are subsequently captured (S1 Fig). The entrance to the clear container is a small hole in the shape of an inverted equilateral triangle with 2 cm sides over which a strip of black 5 cm X 9 cm cloth tape (Bear, Saint-Gobain), with an identical triangle opening, is placed internally over the trap entrance (black side facing into trap). Previous laboratory observations indicated that this black tape reduced exit behaviour of the mosquitoes as, once they were no longer attracted to the sound lure, they displayed escape behaviours which avoided the dark tape. The sound lure is programmable for frequency and volume emitted, whether the frequency is played continually or intermittently (30 s on-off) and has a photo-detector which turns it off during the night to save power. Both the MAST Sticky and MAST Spray versions of this trap were deployed in these trials. The MAST Sticky uses an internal killing chamber and sticky panel to capture mosquitoes whereas the MAST Spray lacks the extra killing chamber and contains insecticide to knock down mosquitoes entering the clear container housing the sound lure. Both versions are described in detail by Staunton, Crawford et al. [18].

### Field sites

**Fig 1:**
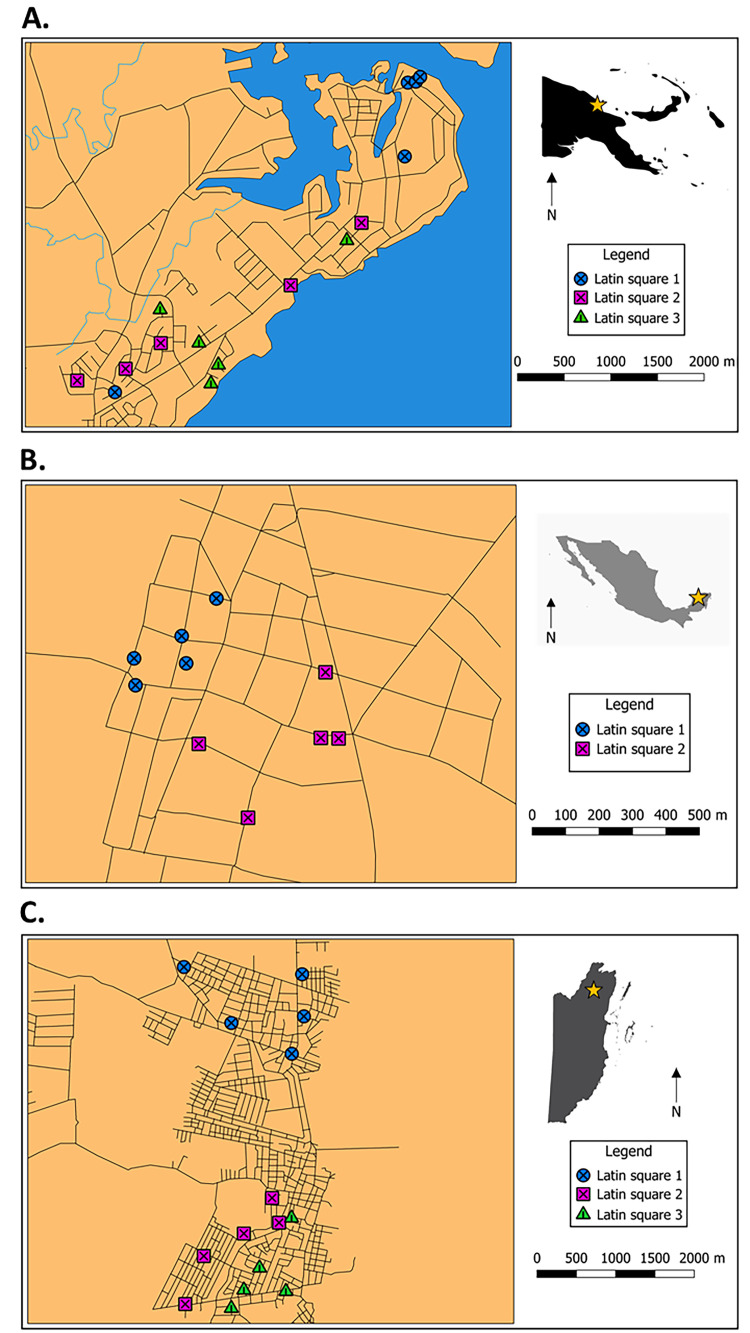
Field sites with trap locations in A) Madang, Papua New Guinea, B) Molas, Mexico and C) Orange Walk Town, Belize. Stars indicate town locations within countries and different symbols indicate different Latin squares specific to each field site. Main maps were created in QGIS 3.4 using layers created by Donovan Leiva and silhouette maps were sourced from vectorstock.com (PNG), dreamstime.com. (Mexico) and freevectormaps.com (Belize).

Three Latin Square trials were run simultaneously within Madang, situated at 3 m elevation and a latitude of 5°S, in Papua New Guinea between 30 May and 31 October 2019 (Fig 1A). Meanwhile, two Latin Square trials were performed between 19 June and 27 September 2019 in the Mexican village of Molas, south of Merida at 10 m elevation and latitude of 20° north, approximately 35 km from the nearest coast (Fig 1B). Lastly, three Latin Square trials were run simultaneously between 3 July and 16 October 2019 in Orange Walk Town (33 m elevation, a latitude of 18° north and approximately 48 km from the nearest coast), Belize, Central America (Fig 1C).

During the experimental period in Madang, the mean monthly air temperatures ranged from 21.6°C to 30.3°C, with 1,970 mm total rainfall and an average humidity of 72% (Madang Airport Weather Services). In Molas, temperatures ranged from 20.6°C to 40.2°C and the total rainfall was 721.6 mm during the trial period (Universidad Autónoma de Yucatán, Merida, México). While in Orange Walk Town during the experimental period, temperatures ranged from 22.2°C to 38.4°C, with an average 73% relative humidity and 343.67 mm total rainfall (Belize Vector & Ecology Center weather station in Orange Walk Town).

Trap locations were generally in secure, sheltered, dark and unobtrusive areas associated with homes (i.e., least interruptive to routine household activities), selected with the homeowner’s consent. Within PNG, these locations were mostly situated near doors, or underneath raised dwellings and in Molas, traps were also positioned with access to mains power (for BGS trap requirements). Trap locations in Molas and Orange Walk Town were often in structures external to the home but within the peridomestic area (e.g., unused pig sty, external cooking structures and sheds). The mean (± S. E.) distances between nearest traps within each town were 317 m (± 56 m), 129 m (± 25 m) and 465 m (± 67 m) for Madang, Molas and Orange Walk Town, respectively.

### Trap settings

Within Madang, each Latin Square trial consisted of five treatments: 1) a BGS trap (unbaited version 2 [42]) powered using car batteries (12 V, 50 Ah; manufactured by Bolt (Guangdong, China) or Yuasa (Kyoto, Japan); and four MAST Sticky versions, with a sound lure set to either 2) 450 Hz, 3) 500 Hz, 4) 550 Hz or 5) 600 Hz. The sound lures (Verily, South San Francisco, USA) were programmed to emit a specific frequency at 60 dB at the trap entrance intermittently (30 s on-off) during daylight hours. MAST Sticky versions, containing yellow sticky panels (Trappit, manufactured by Entosol (Australia) Pty Ltd, Roselands), cut to 50 x 70 mm, were deployed as a capture agent in the MAST head in Madang based on evidence of *Ae*. *aegypti* and *Ae*. *albopictus* pyrethroid-resistance in study area [43]. Traps were operated daily and randomly rotated weekly within their Latin Square. MASTs were serviced weekly, by removing all catches, replacing sticky panels, and checking the sound lure settings. BGS traps operated continuously, with catch bags and batteries replaced twice per week, after 4 days and then again after 3 more days.

Five treatments were also evaluated simultaneously in Molas consisting of one BGS trap (unbaited and connected to mains power) and four MAST Spray versions [24] set to the same four frequencies and sound lure settings as in PNG trials. *Aedes aegypti* were also pyrethroid (deltamethrin) resistant in this region [44] so MAST Spray versions were treated with H24 (Naucalpan, Mexico; active ingredients: propoxur 1.507 g/kg, prallethrin 0.093 g/kg and deltamethrin 0.35 g/kg) known to knock down synthetic pyrethroid resistant mosquitoes [45,46]. All Molas traps operated for a single, 24-hour period each week to conform to standard surveillance protocols in the study area. Traps were randomly rotated within each Latin square among participating households weekly throughout each five-week trial.

In Orange Walk Town we again deployed five treatments for each Latin Square trial including a BGS trap (unbaited and powered using motorbike batteries (12 V, 5 Ah; manufactured by Outdo, Fujian, China) and four MAST Spray versions set to the same four frequencies and sound lure settings as in Madang and Molas. All MASTs used H24 insecticidal spray to kill captured mosquitoes. Traps were left *in situ* for an eight-hour period one day per week beginning between 08:00 and 10:00 and then collected later that day, in the same order they were deployed, between 16:00 and 18:00, conforming to local *Aedes* surveillance protocols for other studies in the area. As in PNG and Mexico, all traps were randomly rotated amongst participating households each week within a single trial until all MAST and BGS traps had been evaluated at each household sampling site (i.e., five weeks total).

For all trial locations, captured mosquitoes were transported to in-country laboratories and identified to species using relevant morphological keys [47,48], sexed and counted by date of capture, sampling site, trap and lure type. For the purposes of this study the term ‘bycatch’ refers to mosquito species other than the two target species (*Ae*. *aegypti* or *Ae*. *albopictus*) as well as all other (non-culicid) invertebrates. All other (non-culicid) invertebrates were sorted to order, counted and their capacity for flight noted (for example, wingless ants were recorded as Formicidae whereas all other Hymenoptera were noted to be ‘Hymenoptera (winged)’).

### Data analysis

All analyses were conducted within the R statistical environment ver 3.5.3 [49] on data supplied (S1 Table). Male *Ae*. *aegypti*, *Ae*. *albopictus* (for Madang only) and *Culex* abundance (count) data were set as response variables and analysed separately for each country. For Madang data, we analysed male *Cx*. *quinquefasciatus* only as other *Culex* species were rarely caught. For the Molas male *Culex* data we combined *Cx*. *quinquefasciatus*, *Cx*. *nigripalpus* and *Cx*. *restuans* abundance data and for the Orange Walk male *Culex* data, we combined *Cx*. *quinquefasciatus* and *Cx*. *restuans* abundance data to assess *Culex* catches in the traps.

We fit the treatment parameter ‘trap-type’ to each response variable by specifying a generalized linear mixed model (GLMM) with a negative binomial distribution and logit link function using the *lme4* package [50]. Initial model runs using Poisson distributions were consistently overdispersed. We included the parameters ‘trap location’ and ‘week’ in the model as random factors to account for any influences on response data between trap locations and throughout time. Trap fails—where the trap was interfered with or, in the case of BGS traps, the battery was depleted—were removed from all analyses. For PNG data, where BGS traps were serviced twice per week, we included an offset parameter in the model to account for trap fails by specifying the number of days (out of seven) the trap was operational. Once models were created, the effect of predictors within each model were analysed using an analysis of deviance in the *car* package [51]. Lastly, post-hoc Tukey test comparisons were used to determine differences among the least-squares means of trap type groups, when significantly differences were found using the *emmeans* package [52].

Trap-type was unable to be fitted to the *Culex* response data from Molas using the above method as there was perfect separation in the data as *Culex* were not caught in the 600 Hz MASTs. Instead we employed a Bayesian generalized linear model, using the *arm* package [53] to fit parameters to this response variable. This package uses the weakly informative Cauchy distribution as the prior distribution and was applied to data with separation issues [54]. GLMMs similar to the above would not run for this data set due to the random effects being too complex to be supported by the data so we instead ran GLMs including ‘trap location’ and ‘week’ as fixed factors to account for the influences between locations and over time on the response data. Significant interactions between fixed factors were not detected for any models and therefore were not reported or further analysed regarding the final least complex adequate models.

To compare the mean rates of positive detections of target species between each trap type abundance data were transformed into binomial data sets by converting all abundance values greater than zero to one. A set of GLM/Ms were then fitted to these new response variables using identical models above except that binomial, rather than negative binomial distributions, were fitted to response data. Again, analyses of deviance and post-hoc Tukey tests were performed to compare differences between groups as described above.

## Results

### Invertebrate catches

In total 28,796 invertebrates were caught from all traps in all countries, including 15,166 mosquitoes and 13,630 other (not-culicid) invertebrates (Table 1).

**Table 1:** Total abundance of taxa caught by trap type in all sites.

Location	Taxa	BGS trap	MAST 450 Hz	MAST 500 Hz	MAST 550 Hz	MAST 600 Hz	Total
Madang, Papua	*Aedes aegypti* male	147	99	81	128	93	548
New Guinea	*Aedes aegypti* female	145	0	0	0	0	145
	*Aedes albopictus* male	118	44	157	210	129	658
	*Aedes albopictus* female	350	0	0	0	0	350
	*Culex annulirostris* male	15	3	0	0	0	18
	*Culex annulirostris* female	11	0	0	0	0	11
	*Culex quinquefasciatus* male	5,857	16	20	12	2	5,907
	*Culex quinquefasciatus* female	4,149	0	0	0	0	4,149
	Other mosquitoes	10	0	0	0	0	10
	Mosquitoes (totals)	10,802	162	258	350	224	11,796
	Other invertebrates	11,530	37	18	50	34	11,669
	Total invertebrates	22,332	199	276	400	258	23,465
Molas, Mexico	*Aedes aegypti* male	147	206	182	229	86	850
	*Aedes aegypti* female	145	0	0	0	0	145
	*Culex quinquefasciatus* male	365	165	142	6	0	678
	*Culex quinquefasciatus* female	119	0	0	0	0	119
	*Culex nigripalpus* male	67	0	0	0	0	67
	*Culex nigripalpus* female	81	0	0	0	0	81
	*Culex restuans* male	182	161	73	24	0	440
	*Culex restuans* female	2	0	0	0	0	2
	Other mosquitoes	11	0	0	0	0	11
	Mosquitoes (totals)	1,119	532	397	259	86	2,393
	Other invertebrates	974	9	5	79	2	1,069
	Total invertebrates	2,093	541	402	338	88	3,462
Orange Walk	*Aedes aegypti* male	125	145	132	112	109	623
Town, Belize	*Aedes aegypti* female	96	1	0	0	0	97
	*Aedes albopictus* male	3	2	2	4	0	11
	*Aedes albopictus* female	7	0	0	0	0	7
	*Culex quinquefasciatus* male	11	0	0	0	0	11
	*Culex quinquefasciatus* female	28	0	0	0	0	28
	*Culex restuans* male	131	23	12	13	1	180
	*Culex restuans* female	20	0	0	0	0	20
	Mosquitoes (totals)	421	171	146	129	110	977
	Other invertebrates	862	9	8	5	8	892
	Total invertebrates	1,283	180	154	134	118	1,869
All locations	**All mosquitoes (totals)**	**12,342**	**865**	**801**	**738**	**420**	**15,166**
	**All other invertebrates**	**13,366**	**55**	**31**	**134**	**44**	**13,630**
	**All invertebrates**	**25,708**	**920**	**832**	**872**	**464**	**28,796**

In Madang, 23,465 invertebrates were caught, with MAST traps capturing ~1–2% of the total invertebrates sampled by BGS traps (Table 1). Of the 11,796 mosquitoes caught in this location: 147 male *Ae*. *aegypti* were sampled in BGS traps and 401 in MASTs, 118 male *Ae*. *albopictus* were caught in BGS traps and 540 in MASTs and lastly, 5,857 male *Cx*. *quinquefasciatus* were caught in BGS traps with 50 caught in MASTs. Additionally, 11,669 other (not-culicid) invertebrates were also captured (S2 Table).

In Molas, a total of 3,462 invertebrates were caught, with MASTs capturing ~4–26% of the total invertebrate catch sampled by BGS traps (Table 1). Of the 2,393 mosquitoes caught here: 147 male *Ae*. *aegypti* were sampled in BGS traps and 703 in MASTs, 365 male *Cx*. *quinquefasciatus* were caught in BGS traps and 313 in MASTs, 67 male *Cx*. *nigripalpus* were caught in BGS traps and 0 in MASTs and lastly, 182 male *Cx*. *restuans* were caught in BGS traps and 258 in MASTs. An additional 1,069 other invertebrates were also captured (S1 Table).

In Orange Walk Town, 1,869 total invertebrates were captured, with MASTs catching ~9–14% of the total invertebrates caught by BGS traps (Table 1). Of the 977 mosquitoes captured in Orange Walk Town: 125 male *Ae*. *aegypti* were captured in BGS traps and 498 in MASTs, 3 male *Ae*. *albopictus* were caught in BGS traps and 8 in MASTs, 11 *Cx*. *quinquefasciatus* were caught in BGS traps and none in MASTs and lastly, 131 male *Cx*. *restuans* were captured in BGS traps and 49 in MASTs. In total, 892 other invertebrates (not-culicid) were also caught (S2 Table).

### Comparisons of *Aedes* mean abundances and positive detection rates per trap type

**Fig 2:**
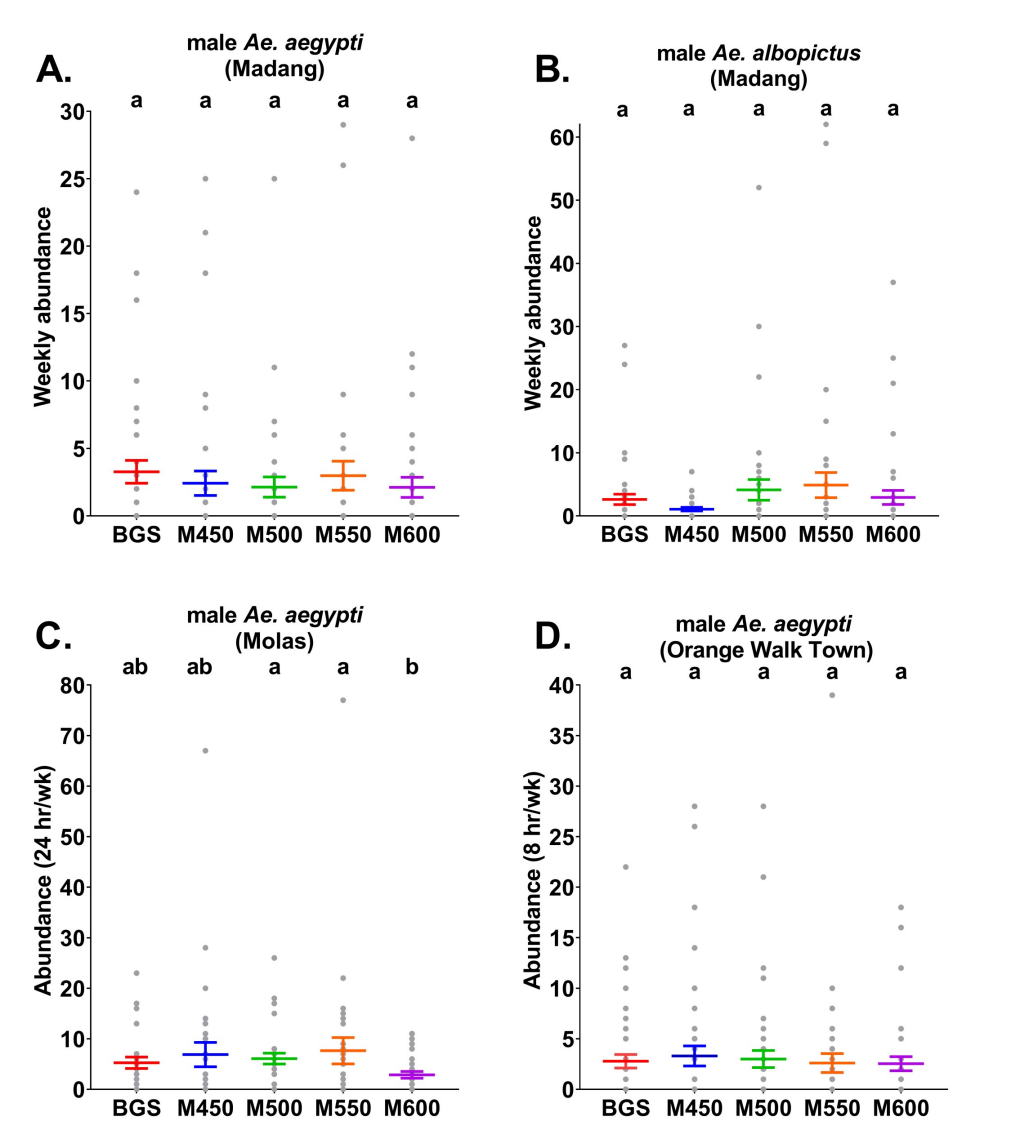
Mean abundance (± S. E.; coloured lines) with raw abundance data (grey points) per trap type of male A) *Ae*. *aegypti* from Madang B) *Ae*. *aegypti* from Molas, C) *Ae*. *aegypti* from Orange Walk Town and D) *Ae*. *albopictus* from Madang. Madang traps were run continuously each week, whereas in Molas traps were operated for a 24 hr period each week and traps in Orange Walk Town were operated for an 8 hr period each week. BGS = BG-Sentinel traps, M450 = MASTs with 450 Hz, M500 = MASTs with 500 Hz, M550 = MASTs with 550 Hz and M600 = MASTs with 600 Hz. Different letters above Molas points indicate significantly different catch rates between treatments determined by models run separately for each target species (males only) and country (Tukey HSD, *P*<0.05).

In Madang, there were no significant differences (ꭓ^2^ = 7.8, *df* = 4, *P* = 0.097, n = 38–45) between the weekly mean abundances of male *Ae*. *aegypti* among treatments (Fig 2A and S3 Table). Additionally, there were no significant differences in the rates of positive detections of male *Ae*. *aegypti* by trap-type (ꭓ^2^ = 3.3, *df* = 4, *P* = 0.5, n = 38–45). Male *Ae*. *aegypti* were positively detected in 47% of the weekly samples from the BGS traps and in 37%, 42%, 40% and 36% of the weekly samples from the MAST 450 Hz, 500 Hz, 550 Hz and 600 Hz treatments, respectively (S4 Table). In addition, no significant differences (ꭓ^2^ = 8.8, *df* = 4, *P* = 0.07, n = 38–45) were found between the weekly mean abundances of male *Ae*. *albopictus* in Madang per trap type (Fig 2B and S3 Table). Nor were any significant differences detected regarding positive detection rates of male *Ae*. *albopictus* between trap types (ꭓ^2^ = 6.5, *df* = 4, *P* = 0.16, n = 38–45). Male *Ae*. *albopictus* were positively detected in 58% of the weekly samples from the BGS traps and in 39%, 50%, 58% and 45% of the weekly samples from the MAST 450 Hz, 500 Hz, 550 Hz and 600 Hz treatments, respectively (S4 Table).

In Molas, unlike Madang, significant differences (ꭓ^2^ = 13.2, *df* = 4, *P* = 0.01, n = 28–30) were noted between the weekly mean abundances of male *Ae*. *aegypti* according to treatment (Fig 2C and S3 Table). Male *Ae*. *aegypti* were caught in higher abundance in MASTs set to 500 or 550 Hz than those set to 600 Hz. Significant differences were also detected between positive detection rates of male *Ae*. *aegypti* for each trap type (ꭓ^2^ = 9.6, *df* = 4, *P* = 0.048, n = 28–30). However, post-hoc Tukey tests (*P* ≤ 0.05) indicated that male *Ae*. *aegypti* were positively detected at higher rates only in the MAST 500 Hz (93%) relative to the MAST 600 Hz traps (60%). Positive detections of these males from weekly samples were recorded at 75%, 70% and 80% from the BGS traps, MAST 450 Hz and MAST 500 Hz treatments, respectively (S4 Table).

In Orange Walk Town, no significant differences (ꭓ^2^ = 0.83, *df* = 4, *P* = 0.93, n = 43–45) were indicated between weekly mean abundances of male *Ae*. *aegypti* (Fig 2D and S3 Table). Positive detection rates of male *Ae*. *aegypti* also did not significantly differ between trap- types (ꭓ^2^ = 2.4, *df* = 4, *P* = 0.65, n = 43–45). Male *Ae*. *aegypti* were positively detected in 56% of the weekly samples from the BGS traps and in 43%, 50%, 53% and 49% of the weekly samples from the MAST 450 Hz, 500 Hz, 550 Hz and 600 Hz treatments, respectively (S4 Table).

### Comparisons of Culex mean abundances and positive detection rates per trap type

**Fig 3:**
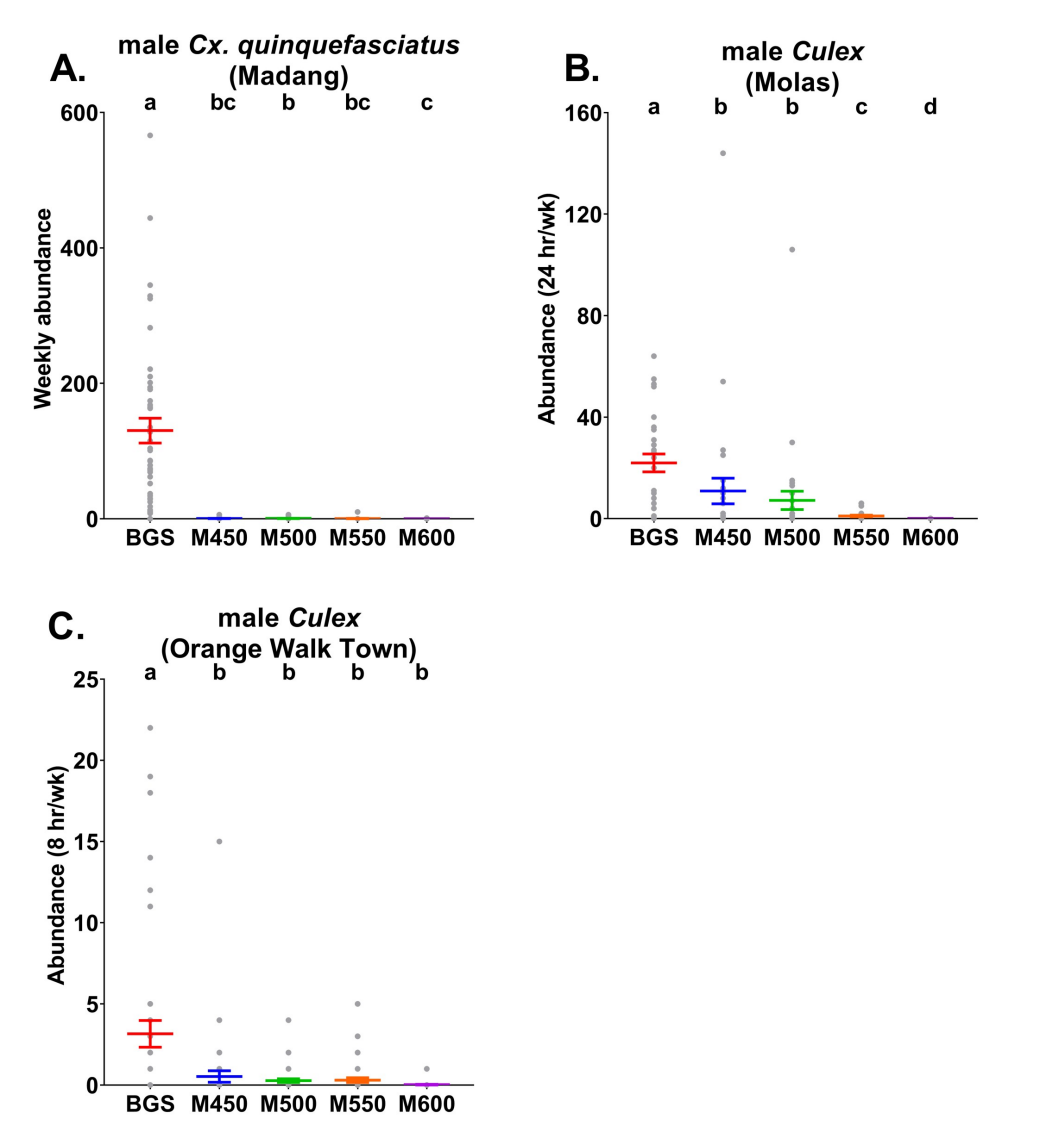
Mean abundance (± S. E.; coloured lines) with raw abundance data (grey points) per trap type of male A) *Cx*. *quinquefasciatus* from Madang B) *Cx*. *quinquefasciatus*, *Cx*. *restuans* and *Cx*. *nigripalpus* combined from Molas and C) *Cx*. *quinquefasciatus* and *Cx*. *restuans* combined from Orange Walk Town. Madang traps were run continuously each week, whereas in Molas traps were operated for a 24 hr period each week and traps in Orange Walk Town were operated for an 8 hr period each week. BGS = BG-Sentinel traps, M450 = MASTs with 450 Hz, M500 = MASTs with 500 Hz, M550 = MASTs with 550 Hz and M600 = MASTs with 600 Hz. Different letters above points indicate significantly different catch rates between treatments determined by models run separately for each target species (males only) and country (Tukey HSD, *P*<0.05).

In Madang, there were significantly more male *Cx*. *quinquefasciatus* (ꭓ^2^ = 625.5, *df* = 4, *P* < 0.05, n = 45) caught weekly in the BGS traps than all MAST frequency versions (Fig 3A and S3 Table). Additionally, there were significantly more male *Cx*. *quinquefasciatus* caught in the MASTs set to 500 Hz than to 600 Hz. The rates of positive detection of male *Cx*. *quinquefasciatus* also significantly differed between BGS traps and all MAST versions (ꭓ^2^ = 37.1, *df* = 4, *P* < 0.05, n = 38–45), but not between MASTs frequencies when analysed using post-hoc Tukey tests (*P* ≤ 0.05). Male *Cx*. *quinquefasciatus* were positively detected in 98% of the weekly samples from the BGS traps and in 15%, 18%, 7% and 5% of the weekly samples from the MAST 450 Hz, 500 Hz, 550 Hz and 600 Hz treatments, respectively (S4 Table).

Male *Culex* mean weekly abundances in Molas (during a 24 hr period each week) significantly differed between trap types (ꭓ^2^ = 144.1, *df* = 4, *P* < 0.05, n = 28–30; Fig 3B and S3 Table). Highest male *Culex* abundance rates were recorded in the BGS traps and, within MASTs, male *Culex* rates declined as the sound lure frequency was increased. Positive detection rates of male *Culex* also significantly differed by trap-type (ꭓ^2^ = 70, *df* = 4, *P* < 0.05, n = 28–30) with BGS traps displaying higher rates than all MAST treatments. There were no significant differences in positive detection rates of male *Culex* between MASTs set to different sound lure frequencies (Tukey, *P* ≤ 0.05). Male *Culex* were positively detected in 93% of the weekly samples from the BGS traps and in 50%, 47%, 40% and 0% of the weekly samples from the MAST 450 Hz, 500 Hz, 550 Hz and 600 Hz versions, respectively (S4 Table). For these models, run with station and week as fixed factors, station was found to significantly influence the number of *Culex* caught per week (ꭓ^2^ = 22.1, *df* = 9, *P* = 0.008, n = 28–30) whereas week did not (ꭓ^2^ = 20.1, *df* = 14, *P* = 0.13, n = 28–30). In relation to positive detection rates of male *Culex* in Molas, neither station (ꭓ^2^ = 7.3, *df* = 9, *P* = 0.6, n = 28–30) nor week (ꭓ^2^ = 17.3, *df* = 14, *P* = 0.23, n = 28–30) significantly influenced the response variable.

BGS trap mean weekly abundances of male *Culex* (caught over an 8 hr period each each) in Orange Walk Town were significantly greater (ꭓ^2^ = 58.2, *df* = 4, *P* < 0.05, n = 43–45) than those from all MAST treatments (Fig 3C and S3 Table). Male *Culex* positive detection rates also significantly varied (ꭓ^2^ = 31.4, *df* = 4, *P* < 0.05, n = 43–45). The BGS traps displayed higher rates of positive detection for male *Culex* (51%) than all MAST treatments which were positive in 11%, 16%, 14% and 2% of all weekly samples from the MAST 450 Hz, 500 Hz, 550 Hz and 600 Hz traps, respectively (S4 Table).

## Discussion

The MASTs evaluated in the current study specifically and effectively captured male *Ae*. *aegypti*, relative to BGS traps, when set to various frequencies in one Pacific and two Latin American dengue endemic countries. These findings not only reinforce the utility of this sound trap for surveillance of male *Ae*. *aegypti*, but also as a suitable platform for continued development of a smart sensor system, which only needs to process a limited suite of species.

These results also suggest that male *Ae*. *aegypti* respond positively to a range of female wingbeat frequencies in natural environments. The wingbeat frequencies displayed by females may vary due to a range of factors such as age, size and even ambient temperature [27,32–36]. Field populations of *Ae*. *aegypti* may therefore contain substantial heterogeneity in the wingbeat frequencies produced by females and positive male responses across a range of frequencies may contribute to enhancing the probability of successful reproduction [27]. Whether our *Ae*. *aegypti* male catch rates in MASTs is due to individual males positively responding to a range of frequencies or similar proportions of males within a field population positively responding to different frequencies is unclear. Additionally, the precise influence, if any, of varying ambient temperatures on male capture rates in sound traps would also require further investigation. However, this range in attractive frequencies offers flexibility in sound trap frequency selection which could be potentially useful for the further reduction of bycatch.

Our findings also indicated that male *Culex* were most attracted to MASTs set to lower frequencies in all study localities and male *Ae*. *aegypti* were less abundant in MASTs set to the highest frequency in Molas. Additionally, male *Ae*. *albopictus*, which were caught in comparable rates in all trap-types, peaked in abundance in MASTs set to 550 Hz. Although female wingbeat frequencies can vary widely due to many factors, previous studies have reported female *Culex* wingbeat frequencies to be generally below 400 Hz whereas female *Ae*. *aegypti* wingbeat frequencies tend to be a little higher, with means such as 458–460 Hz, and female *Ae*. *albopictus* wingbeat frequencies are higher still, with means of 536–544 Hz, for example [22–24].

The preference of *Culex* for the lower frequency lures in MASTs in the current study is consistent with Ikeshoji and Ogawa [39] who demonstrated an affinity of *Culex* to their sound traps set at 400 Hz. Our decline of male *Ae*. *aegypti* mean abundance in Molas with traps set to 600 Hz confirmed earlier work by Johnson and Ritchie [14], in which free-flying male *Ae*. *aegypti* catch rates in semi-field experiments were highest in Gravid *Aedes* Traps (Biogents, Regensburg, Germany) with sound lures set to 484 Hz compared to those set to 560 Hz or 715 Hz.

This study not only confirms previous findings that the MAST captures comparable mean abundances and positive weekly detection rates of male *Ae*. *aegypti* to those caught in BGS traps [18], but also extends this work to include similar findings regarding male *Ae*. *albopictus*. Our results in Madang indicated that catches of male *Ae*. *albopictus* decrease in sound traps set to frequencies below 500 Hz which is consistent to field studies performed in northern Australia by Swan, Russel et al. [41]. Balestrino, Iyaloo et al. [12] investigated the attraction of male *Ae*. *albopictus* to 545 Hz, 600 Hz and 649 Hz, as well as a frequency-sweep ranging between 500–650 Hz, within a climatic chamber using a prototype sound trap. While, similar to us, they did not find any significant differences in male attraction to traps set at fixed frequencies, they recorded a light decline in the number of males attracted to 650 Hz, relative to 600 Hz, whereas our mean male abundances began to decline at 600 Hz, relative to 550 Hz [12] and the mean abundances of male *Ae*. *albopictus* sampled by Swan, Russel et al. [41] declined between 650 Hz and & 700 Hz. Such differences in the upper limits of male attraction may reflect variations between male *Ae*. *albopictus* of different strains. Balestrino, Iyaloo et al. [12] also demonstrated significantly greater male catch rates in sound traps set with the frequency-sweep, relative to the fixed frequencies, which they attributed to better representing either the range of sounds potentially displayed during swarming or a female in flight, thereby exciting males more. Additionally, they hypothesised that males may experience desensitisation to a fixed frequency and become less responsive. While we set our sound lures to intermittent tones (30 s on-off) to save energy and reduce potential male desensitisation, future field trials with the MAST should also test male mosquito responsiveness using frequency-sweeps.

Regarding bycatch, it is important to note that BGS traps are often deployed with a variety of chemical lures which often significantly increase *Ae*. *aegypti* and *Ae*. *albopictus* catch rates [55–58]. However, how these chemical lures influence bycatch abundance in BGS traps is unknown. Furthermore, the addition of chemical lures to sound traps has been rarely investigated. Staunton, Rohde et al. [59] found that Gravid *Aedes* Traps with sound lures and BG-Lures (Biogents, Regensburg, Germany) did not catch higher abundances of male *Ae*. *aegypti* than those set without these chemical lures in northern Australia. Kanda, Cheong et al. [40] found that the addition of dry ice and a guinea pig to their sound traps greatly increased male *Mansonia* capture rates in Malaysia. Future trials, assessing the efficacy of the MAST, should investigate mosquito and other invertebrate catch rates in both MASTs and BGS traps set with chemical lures.

The MAST was designed to capture male *Aedes* mosquitoes by attracting them from a distance with its large black base and then enticing them into the clear capture container with the sound lure, with a physical design which enhances the species-specificity of catches [18]. With the lure set at 60 dB at the trap entrance to avoid irritating people living nearby, the sound lure is only effective over short distances as sound is detected by mosquitoes as particle motion which reduces rapidly with distance [60]. The MAST was not designed to sample *Culex* mosquitoes, especially species which inhabit houses such as those from the *Culex pipiens* complex, which may co-locate with *Ae*. *aegypti*. As such it was unsurprising that male *Culex* were caught in lower abundances, and detected less frequently, in MASTs than BGS traps at all locations. Unlike males of *Ae*. *aegypti* and *Ae*. *albopictus*, male *Culex pipiens* generally swarm over large objects such as trees and buildings, although swarming can occur at ground level [61]. The very low catch rates of *Cx*. *quinquefasciatus* in the MASTs, relative to the BGS traps in PNG, suggest that this strain may not be attracted to the MAST base. However, *Culex pipiens quinquefasciatus*, sourced from The Gambia and maintained in a laboratory in England were reported to have swarmed within a cage over a black marker and adjusted their flight behaviour relative to a non-localised frequency played between 500 and 600 Hz [62]. It is therefore feasible that the *Culex* captured in Mexico were either attracted to the MAST base as a swarm marker and then entered the trap at certain frequencies or were simply at high abundances and responded positively to the sound lures as they randomly intercepted the MASTs. While not designed to catch *Culex*, the MAST could potentially use sound lures run at lower frequencies than those tested in Molas or be physically reconfigured, although potentially at the expense of *Aedes* capture effectiveness, to expand the application of MASTs to monitoring *Culex*.

In light of the above findings and in relation to *Aedes* surveillance, 550 Hz may be the optimal frequency for this MASTs sound lure. At this frequency both male *Ae*. *aegypti* and *Ae*. *albopictus* mean abundances and positive detection rates were effective, relative to the BGS trap, while male *Culex* catch rates were consistently low. These results support previous findings [18] that the MAST is a highly species-specific trap, relative to the BGS trap. Large reductions in bycatch saves significant time and labour for surveillance programs of *Ae*. *aegypti* or *Ae*. *albopictus*. Thus, using a sound lure to capture mosquitoes may enable the development of a cost-effective smart trap to accurately identify catches with a reduced workforce requirement.

## Conclusion

This study is the most extensive reported set of surveys investigating male mosquito capture rates in sound traps set at different frequencies under natural conditions and presents data vital to the effective deployment of sound traps in control programs, such as those mass rearing and releasing *Wolbachia*-infected males. MASTs utilising sound lures set to 450–550 Hz consistently caught male *Ae*. *aegypti* at comparable rates to BG-Sentinel traps in all study locations. Results suggest that MASTs should be set at 550 Hz for male *Ae*. *aegypti* and *Ae*. *albopictus* surveillance in these regions to ensure sensitive detection of the *Aedes* vectors with limited bycatch, including male *Culex*. Our findings will further enable development of a cost-effective smart trap to assist in rigorously monitoring key mosquito vector species all the while reducing burden in person-time.

## Supporting information

S1 Fig The MAST deployed in A) a laundry area in Madang and kitchen areas in B) Molas and C) Orange Walk Town.
(TIF)

S1 Table Male mosquito data from each country on which analyses were performed. (XLSX)

S2 Table Total abundance of other invertebrates (not Culicidae) caught by trap type in all sites. (XLSX)

S3 Table Mean weekly (± S. E.) abundances of target mosquito species (males only) by trap type in all sites. Note in Madang traps were run continuously each week, whereas in Molas traps were operated for a 24 hr period each week and in Orange Walk Town traps were operated for an 8 hr period each week. *Culex* spp. from Molas are comprised of combined abundance values for *Cx*. *quinquefasciatus*, *Cx*. *restuans* and *Cx*. *nigripalpus* and *Culex* spp. from Orange Walk Town consist of combined abundance values for *Cx*. *quinquefasciatus* and *Cx*. *restuans*.
(XLSX)

S4 Table Mean weekly (± S. E.) proportions of positive detection rates of target mosquito species (males only) by trap type in all sites. Note in Madang traps were run continuously each week, whereas in Molas traps were operated for a 24 hr period each week and in Orange Walk Town traps were operated for an 8 hr period each week. *Culex* spp. from Molas are comprised of combined abundance values for *Cx*. *quinquefasciatus*, *Cx*. *restuans* and *Cx*. *nigripalpus* and *Culex* spp. from Orange Walk Town consist of combined abundance values for *Cx*. *quinquefasciatus* and *Cx*. *restuans*.
(XLSX)
