# Supplementary figures and images for: Outcomes from international field trials with Male Aedes Sound Traps: Frequency-dependent effectiveness in capturing target species in relation to bycatch abundance
Source: PLoS Negl Trop Dis. 2021 Feb 25;15(2):e0009061. doi: 10.1371/journal.pntd.0009061 (PMC7906331; doi:10.1371/journal.pntd.0009061)

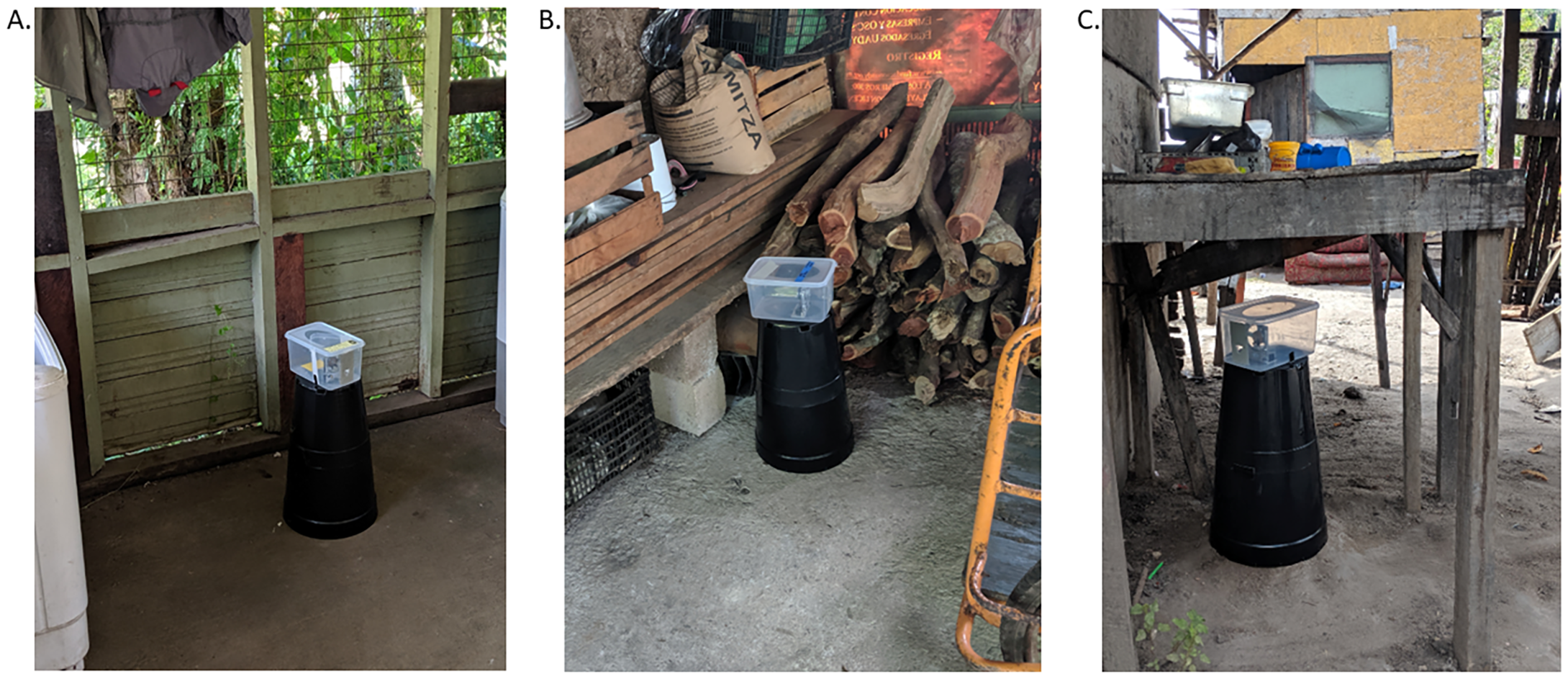

Supplement: S1 Fig — The MAST deployed in A) a laundry area in Madang and kitchen areas in B) Molas and C) Orange Walk Town. (TIF) [file pntd.0009061.s001.tif]
